# Autoimmunity to Sphingosine-1-Phosphate-Receptors in Systemic Sclerosis and Pulmonary Arterial Hypertension

**DOI:** 10.3389/fimmu.2022.935787

**Published:** 2022-07-04

**Authors:** Hans Gluschke, Elise Siegert, Waldemar B. Minich, Julian Hackler, Gabriela Riemekasten, Wolfgang M. Kuebler, Szandor Simmons, Lutz Schomburg

**Affiliations:** ^1^ Institute for Experimental Endocrinology, Charité – Universitätsmedizin Berlin, Berlin, Germany; ^2^ Department of Rheumatology and Clinical Immunology, Charité – Universitätsmedizin Berlin, Berlin, Germany; ^3^ Berlin Institute of Health at Charité – Universitätsmedizin Berlin, Berlin, Germany; ^4^ Department of Rheumatology, University Medical Center Schleswig Holstein, Lübeck, Germany; ^5^ Institute of Physiology, Charité – Universitätsmedizin Berlin, Berlin, Germany; ^6^ Deutschs Zentrum für Herz-Kreislauf-Forschung e.V. (DZHK) (German Centre for Cardiovascular Research), Partner Site Berlin, Berlin, Germany

**Keywords:** autoantibodies, G-protein coupled receptor, autoimmune disease, immunoglobulin, rheumatology, immunology, sphingolipid, sphingosine-1-phosphate

## Abstract

**Context:**

Pulmonary arterial hypertension (PAH) is a frequent extracutaneous manifestation of systemic sclerosis (SSc). PAH is characterized by increased vasomotor tone, progressive remodeling of pulmonary arteries and arterioles, consequentially increased pulmonary vascular resistance, right heart hypertrophy, and eventually right ventricular failure. Autoimmunity against G-protein coupled receptors (GPCRs) has been implicated in the development of SSc-associated PAH. Sphingosine-1-phosphate (S1P) receptors (S1PR) present a potential, yet so far untested antigen for PAH autoimmunity, given the documented role of S1P/S1PR signaling in PAH pathogenesis.

**Objective:**

We hypothesized that S1P receptors (S1PR) may constitute autoantigens in human patients, and that the prevalence of autoantibodies (aAb) to S1PR1, S1PR2 and S1PR3 is elevated in SSc patients and associated with PAH.

**Methods:**

For this exploratory study, serum samples from 158 SSc patients, 58 of whom with PAH, along with 333 healthy control subjects were screened for S1PR-aAb. S1PR1-3 were expressed as fusion proteins with luciferase in human embryonic kidney cells and used to establish novel *in-vitro* assays for detecting and quantifying S1PR-aAb. The fusion proteins were incubated with serum samples, the aAb-S1PR complexes formed were precipitated by protein-A, washed and tested for luciferase activity. Commercial anti-S1PR-antibodies were used to verify specificity of the assays.

**Results:**

All three assays showed dose-dependent signal intensities when tested with S1PR-subtype specific commercial antibodies. Natural aAb to each S1PR were detected in healthy controls with a prevalence of <10% each, i.e., 2.7% for S1PR1-aAb, 3.6% for S1PR2-aAb, and 8.3% for S1PR3. The respective prevalence was higher in the cohort of SSc patients without PAH, with 17.1% for S1PR1-aAb, 19.0% for S1PR2-aAb, and 21.5% for S1PR3. In the subgroup of SSc patients with PAH, prevalence of aAb to S1PR2 and S1PR3 was further elevated to 25.9% for S1PR2-aAb, and 27.6% for S1PR3. Notably, the majority of patients with positive S1PR2-aAb (60.7%) or S1PR3-aAb (71.9%) displayed interstitial lung disease.

**Conclusion:**

S1PR1–3 can constitute autoantigens in humans, particularly in SSC patients with PAH. The potential pathophysiological significance for the etiology of the disease is currently unknown, but the elevated prevalence of S1PR2-aAb and S1PR3-aAb in SSC patients with PAH merits further mechanistic investigations.

## Introduction

Pulmonary hypertension (PH) is characterized by a mean pulmonary arterial pressure (mPAP) of ≥ 20 mmHg at rest. Its clinical features may gradually progress from an initial asymptomatic course to dyspnea and orthopnea, and eventually to right heart hypertrophy, failure and death. The pathophysiology of PH is characterized by vascular remodeling, endothelial dysfunction and increased vascular tone, predominantly in small to medium-sized pulmonary arterioles (1). Precapillary pulmonary arterial hypertension (PAH) constitutes the first group in the 2018 consensus on the clinical classification of PH into five groups (2). ‘PAH’ is diagnosed when mPAP at rest is measured ≥ 20 mmHg, yet pulmonary artery wedge pressure is ≤ 15 mmHg and other causes of pre-capillary PH (e.g. lung disease or chronic thromboembolic pulmonary hypertension) are excluded (3). The subcategory PAH can be specified further by etiology into idiopathic, hereditary, drug- and toxin-induced forms of PAH, or PAH associated with connective tissue disease, HIV or congenital heart disease (4). While essential pathophysiological elements have been elucidated over the past decades, the etiology of PAH remains incompletely understood (5). Besides an epigenetic dysregulation, alterations in bone morphogenetic protein signaling, abnormalities in mitochondrial metabolism, and dynamic inflammatory, autoimmune processes contribute to the pathogenesis of PAH (6). Specifically, cell-based and autoantibody (aAb) related immune dysregulation have been implicated in the development of PAH, predominantly with respect to idiopathic and connective tissue disease-associated PAH (7). Antinuclear Ab (ANA) serve as diagnostic hallmark of connective tissue disease, and specific ANA are associated with an increased risk for PAH. In addition, aAb to certain G-protein coupled receptors (GPCR) such as angiotensin 1-receptor or endothelin receptor-1 have recently been associated with PAH, and may promote pathological vasoconstriction and vascular remodeling by acting as agonists of the respective GPCR (8). Another line of research has recently identified signaling *via* the small bioactive lipid mediator sphingosine-1-phosphate (S1P) and its receptors (S1PR), which regulate vasoconstriction, fibrosis, and lymphocyte trafficking (9–11) as potential pathomechanism in PAH.

S1P can be generated at the inner layer of the cell membrane from its sphingolipid precursor sphingosine by sphingosine-kinase (SPHK)-1 or -2 *via* the specific S1P-transporters major facilitator superfamily domain-containing protein 2B (MFSD2B, in erythrocytes and platelets) (12), or spinster-homologue-2 (SPNS2, in blood and lymphatic endothelial cells) S1P can then be released into the extracellular space and the circulation (13, 14). Extracellular S1P can bind and activate five different human S1P-receptors (S1PR), namely S1PR1 to S1PR5, all of which belong to the superfamily of GPCR (15, 16). Besides the crucial role of S1PR1, S1PR2 and S1PR3 in the maturation, activation and chemotaxis of immune cells (11, 17), these ubiquitously expressed S1PR are also the major receptor subtypes in the cardiovascular system with high expression in pulmonary artery smooth muscle cells (PASMC) (18). Of note, S1PR1- and S1PR3-signaling plays an important role in preserving vascular functions and blood pressure homeostasis by controlling endothelial nitric oxide synthase (eNOS)-derived nitric oxide (NO) production, which underlies the anti-hypertensive effect of S1P (19). However, elevated levels of SPHK1 in PASMC result in an autocrine “inside-out” S1PR-signaling that can stimulate PASMC proliferation *via* S1PR2, which has been proposed appears upregulated in PASMC of idiopathic PAH patients (9, 15). In parallel, activation of S1PR2 (and potentially also S1PR4) in PASMC causes pulmonary vasoconstriction (20–22). In combination, the effects of S1P on pulmonary arterial endothelial and smooth muscle cells emphasize the importance of a tightly controlled S1P/S1PR-signaling in vascular homeostasis that when impaired can drive vasoconstriction, vascular remodeling, and endothelial dysfunction, which increase pulmonary vascular resistance and mPAP ultimately resulting in the development of PAH. Experimental proof-of-principle for this concept was demonstrated by the fact that genetic deficiency or pharmacological inhibition of either SPHK1 or S1PR2 effectively attenuated the development of PH in rodent models of chronic hypoxic PH (9). Further, a specific role of S1PR2 and S1PR3 has been described in fibrosis, in particular in relation to inflammation and tissue injury, leading to cell death, matrix deposition and finally end organ dysfunction (23). Due to the crucial role of S1P-signaling in homeostasis of the immune system and the endothelium, specific S1PR-modulators are already used in autoimmune disease such as multiple sclerosis and inflammatory bowel disease and constitute a promising therapeutic option for rheumatoid arthritis, systemic lupus erythematosus or systemic sclerosis (SSc) (24–26).

Systemic sclerosis, as one of the rheumatic diseases with the highest mortality, is also characterized by autoimmune dysregulation, endothelial dysfunction and chronic inflammation (27, 28). In more than 90% of patients, ANA are detected, constituting an important cornerstone of the diagnosis and supporting the classification of SSc as an autoimmune disease (29). Additional aAb to other members of the GPCR superfamily have recently been described in SSc by us and others (28, 30). The complication of PAH on a background of SSc (SSc-PAH) affects up to 12% of patients with SSc, constituting the major cause of death in SSc. Still, diagnostic and prognostic markers for PAH are few, therapeutic measures remain poorly effective and treatment options are limited, causing physicians to pursue symptomatic approaches rather than curative strategies (31, 32). Besides established immunosuppressive therapies, S1P modulators such as cenerimod already showed promising results in a mouse model of bleomycin-induced fibroses (33).

In view of the ubiquitous function of S1P/S1PR1/eNOS signaling in the vasculature, the established role of S1P/S1PR2 signaling in PASMC proliferation, contraction, and the development of PH, and the specific contribution of S1PR2 and S1PR3 to disease-related extracellular matrix deposition and tissue fibrosis, we speculated that any alterations to the function of one of these three S1PR may be associated with SSc, in particular with respect to PAH development. To this end, we established as a first step novel *in vitro* assays for detection and quantification of aAb binding to S1PR1, S1PR2 or S1PR3, and compared the prevalence of these GPCR-specific aAb in serum samples from healthy controls and SSc patients with or without PAH. Our results indicate an elevated prevalence of S1PR-specific aAb in SSc, in particular for S1PR2-aAb and S1PR3-aAb in patients with SSc-PAH.

## Materials and Methods

### Human Samples From Healthy Controls and SSc Patients

An explorative study was conducted on the prevalence of S1PR-aAb. To this end, a set of commercially available serum samples (n=303) from subjects with a self-assessed status as ‘healthy’ (HC; healthy controls) served as reference (in.vent Diagnostica GmbH, Hennigsdorf, Germany). An additional set of 30 serum samples from healthy subjects were collected at the Rheumatology Department in Lübeck, Germany. Patients suffering from SSc with or without PAH were identified and enrolled into the study at the Rheumatology Department at the Charité – University Medicine Berlin, Berlin, Germany, or at the University Hospital Lübeck, Schleswig Holstein, Germany, in the time period from Nov. 2004 to Dec. 2019. The final cohort consisted of n=158 serum samples from SSc patients, n=58 of which had an additional diagnosis of PAH. Samples were stored at -80°C until transfer to the analytical laboratory in Berlin, and provided to the scientists conducting the laboratory analyses in a blinded fashion. All patients provided their written informed consent for enrollment into the study after detailed explanation of purpose, procedures (blood sampling and analysis) and right to withdraw from participation at any time point. The study was conducted in accordance with the Declaration of Helsinki. Ethical counselling was provided by the Charité Medical School Berlin (10/30/2017, EA1/178/17) and the Board of Ethics of the University of Cologne (#04-037). Baseline characteristics of patients and healthy controls are displayed in **Table 1**.

**Table 1:** Baseline characteristics of the study cohorts.

	Rheumatology Departments Univ. of Lübeck & Charité Berlin		In.vent Diagnostica
Diagnosis	Systemic sclerosis n=158	Healthy controls n=30	Healthy controls n=303
**Sex**			
Female, n (%)	116 (73.9%)	19 (63.3%)	171 (56.4%)
Male, n (%)	41 (26.1%)	11 (36.7%)	132 (43.6%)
**Age**, median (range) [y]	63 (26–84)	52 (22–59)	32 (19–63)
**BMI**, median (range) [kg/m2]	24 (16–48)	–	–
**Disease duration**, median (range) [months]	84 (0–588)	–	–

Missing values were excluded from the calculation.

### Construction of the Receptor–Luciferase Fusion Proteins for Autoantibody Detection

The construction of these novel assays followed an established path, whereby the full open reading frames of the human coding sequences of the three S1PR were individually fused in frame to a luciferase (Luc) reading frame. The resulting receptor-luciferase fusion proteins served as autoantigen baits for autoantibody detection. Briefly, the open reading frames of the human S1PR1, S1PR2 and S1PR3 were synthesized by a commercial supplier (BioTeZ, Berlin-Buch GmbH, Berlin, Germany), each containing suitable restriction sites for directed insertion into a eukaryotic expression plasmid. The strategy is similar to the recently generated assays for the insulin-like growth factor receptor 1, the thyroid hormone transporters MCT8 and MCT10, the GPCR for gonadotropin-releasing hormone, luteinizing hormone or follicle-stimulating hormone or the iodide transporters sodium-iodide symporter and pendrin (34–38). The stop codons were each replaced by a sense codon in order to enable read-through. The expression plasmid backbone pSP-Luc+NF was obtained from Promega (Promega GmbH, Walldorf, Germany). DNA-sequencing for verification of the correct expression cassettes was conducted by LGC Genomics (LGC Genomics GmbH, Berlin, Germany).

### Stable Expression of Fusion Proteins in Human Embryonic Kidney Cells

In order to achieve stable and reproducible expression of the fusion proteins, human embryonic kidney cells (HEK 293) were transfected with the expression vectors and cultured in DMEM/F12 supplemented with 10% fetal bovine serum (FBS) and 1% penicillin/streptomycin. Two days after transfection, 0.8 mg/mL G418 (geneticin, Sigma-Aldrich GmbH, Steinheim, Germany) was added to the medium, and stable clones were selected by the criteria of robust cell growth characteristics and high luciferase activity. Selected clones were expanded and seeded on 165 cm² cell culture dishes for recombinant protein production in medium supplemented with 0.2 mg/dL G418.

### Preparation of Cell–Extracts for Autoantibody Tests

After reaching confluency of 80% or more, stable HEK293 cells expressing the fusion protein of choice were harvested with a cell scraper and collected by centrifugation (10 min, 1000 g, 4°C). Cell pellets were washed twice with pre-cooled PBS and collected again by centrifugation. The resulting pellet was resolved in collection buffer (50 mM Tris-HCl, 100 mM NaCl, 10% glycerol, 0.01% NaN_3_ in dH_2_0) and subsequently lysed by adding 1% Triton X-100 (Sigma-Aldrich). After gentle shaking and final centrifugation (15 min, 4000 g, 4°C), the supernatants were tested for luciferase activity. The resulting cell extracts were diluted in collection buffer to achieve signal intensities of > 1*10^6^ relative light units (RLU) per measurement and stored in aliquots at -80°C until use.

### Quantitative Analysis of Antibodies Binding to Human S1P Receptors

For the quantitative analysis of antibodies reactive with S1P receptors, the cell extracts containing S1PR-Luc fusion proteins were diluted in reaction buffer (50 mM Tris-HCl, 100 mM NaCl, 10% glycerol, 5% milk powder, 5% glucose, 1% Triton X-100, 0.005% NaN_3_ in dH_2_O) to a concentration equivalent to providing 2-4*10^5^ RLU per reaction (Automat Plus LB953, Berthold Technologies, Bad Wildbad, Germany). Each measurement was conducted by incubating 40 µL of diluted cell extract overnight at 4°C with 10 µL of serum sample diluted 1:1 (v/v) with serum buffer (50% glycerol, 100 mM NaCl, 50 mM Tris-HCL, 0.01% NaN_3_ in dH_2_O). After 24 h, an aliquot of 40 µL of protein-A sepharose (ASKA Biosciences GmbH, Berlin, Germany) diluted in reaction buffer (20% v/v) was added to capture the fusion-protein-aAb-complexes that had formed overnight. The samples were centrifuged (1000 g, 2 min, 4°C) and pellets were washed five times using 200 µL Tris-based buffer (50 mM Tris-HCl, pH 7.4, 100 mM NaCl, 10% glycerol, 0.5% Triton X-100 in dH_2_O). After resuspension, luciferase activity was determined by injection of luciferase substrate, signal intensities were measured as RLU with a luminometer (Automat Plus LB953 or Mithras LB943), and used to calculate specific binding indices (BI) as factor above background noise.

### Statistical Analysis

Under the assumption of less than 50% of positive samples in the analysis, average background noise of negative samples was determined for each experimental run in 96-well plates by calculating the mean RLU from the lower half of the obtained signals. This signal was defined as binding index one (BI = 1.0). For analytical comparison, all signals [RLU] were divided by this background signal [RLU] and expressed as BI, denoting the signal strength as times background signal. In order to determine the cut-off for S1PR-aAb-positve samples, a mathematical outlier criterion was used. To this end, a threshold was calculated representing the BI of the 75^th^ percentile of all signals plus 1.5-times the inter quartile range (P75 + 1.5 x IQR). In this manner, a separation of aAb-positive from aAb-negative samples was achieved. All statistical analyses were performed using GraphPad Prism v8.4.0 (GraphPad Software Inc., San Diego, CA, USA). Intra-assay coefficients of variation were determined from repeated measurements of the same positive samples. Tests for normal distribution (D’Agostino-Pearson test, Shapiro-Wilk test, Kolmogorov-Smirnov test) showed a non-parametric distribution of S1PR-aAb-positivity and of the clinical parameters. Group comparisons of continuous clinical characteristics were analyzed for significant differences with two-sided Mann-Whitney test. Binary variables were compared with one-sided (expected direction of difference) or two-sided (explorative hypothesis) Chi Square test for statistical significance, as indicated. Significance was assumed when the p-value was below 0.05; however, the p-values may not be interpreted as confirmative as all analyses were considered exploratory and not adjusted for multiple testing.

## Results

### Establishment of Quantitative Tests for Measuring Autoantibodies to the S1P Receptors

**Figure 1:**
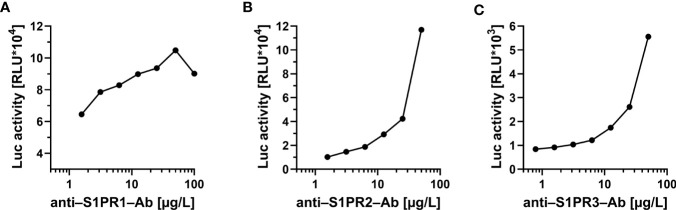
Proof of concept for the novel tests to quantify commercial antibodies to the S1P-receptors. Signal development was tested with commercial antibodies to **(A)** S1PR1, **(B)** S1PR2 and **(C)** S1PR3, respectively. In all three newly developed detection assays, a concentration-dependent decline of signal intensity (expressed as relative light units; RLU) was observed in dilution experiments of commercial receptor-specific antibodies. No cross-reactivity to other receptors was observed. Control measurements were conducted using either monoclonal (to S1PR1 and S1PR2) or polyclonal (to S1PR3) antibody preparations.

Stable HEK293 cell clones were established expressing human S1PR1-, S1PR2- and S1PR3-luciferase (S1PR1-Luc, S1PR2-Luc, S1PR3-Luc) fusion proteins, respectively. Recombinant expression of the fusion proteins to be used as bait in the aAb analyses yielded comparable luciferase activities per preparation. To verify specificity, commercial antibodies recognizing human S1PR1, S1PR2 or S1PR3 were obtained and used as positive standards. Each of the commercial antibodies produced strong signals in the respective assay. Intra-assay coefficients of variation were 14.3% for the S1PR1-, 6.6% for the S1PR2- and 5.3% for the S1PR3-assay. Stepwise dilution experiments using commercial antibodies revealed concentration-dependent signals for all three aAb assays (**Figure 1**).

### Stability of S1PR–aAb in Serum Samples Upon Freezing and Thawing

**Figure 2:**
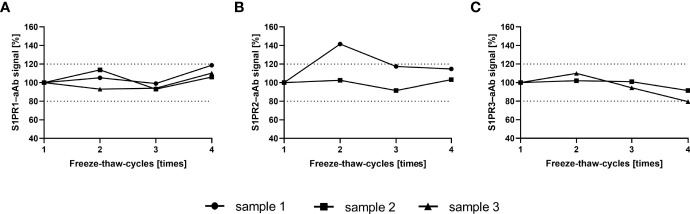
Signal stability upon multiple freeze-thaw-cycles. S1PR–aAb signal stability of aAb-positive serum was assessed with selected samples in the newly generated assays for **(A)** S1PR1–aAb, **(B)** S1PR2–aAb, and **(C)** S1PR3–aAb assay. Samples were subjected consecutively to four freeze and thaw cycles, and all measurements with one exception displayed signal intensities within a 20% range from the initial signal strength determined upon first thawing.

To ensure that freezing and thawing does not interfere with the analysis of S1PR-aAb in human serum, selected samples that had been screened positive for S1PR–aAb were tested after one to four consecutive freeze and thaw cycles. Freezing was achieved on dry ice at –80°C and thawing by standing without agitation at room temperature until completely thawed. The signal intensities remained relatively unaffected by the repeated freezing and thawing, and final signal strength after four rounds of freezing and thawing was within 20% of the initial values determined after the first thawing in all three analytical assays (**Figure 2**). The results support the suitability of frozen human serum samples for assessing aAb to the S1PR by the newly generated analytical tests.

### Prevalence of S1PR–aAb in SSc Patients Versus Healthy Controls

**Figure 3:**
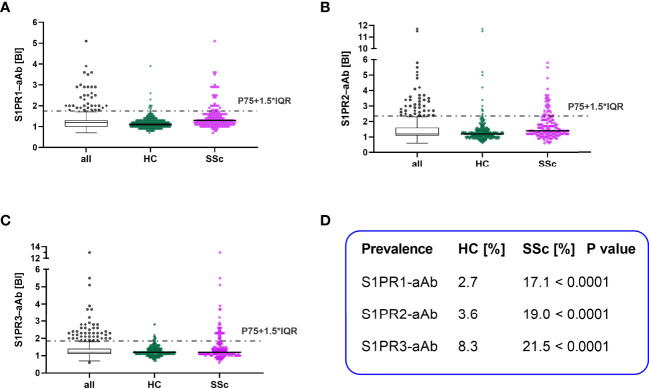
Comparison of S1PR–aAb in healthy controls versus patients with systemic sclerosis. The presence of aAb to the three human S1PR (1–3) was determined in the group of healthy controls (HC) and patients with systemic sclerosis (SSc), and the relative prevalence was compared between both groups. Thresholds are indicated by dotted lines, and values are displayed as scattered dot plots. Black lines represent the median. Whiskers of the box plots denote P25 and P75. Results from HC are denoted in green and of SSc in pink. The prevalence for **(A)** S1PR1–aAb, **(B)** S1PR2–aAb, and **(C)** S1PR3–aAb was elevated in the patient cohort as compared to healthy controls. **(D)** A statistical comparison of SSc and HC for S1PR-aAb was conducted by two-sided Chi square test and revealed significant group differences.

A cohort of serum samples from patients with SSc (n=158) and a collection of serum samples from healthy controls (n=333) was analyzed for natural aAb to S1PR by the three receptor-specific tests. The signals (RLU) were converted into relative binding indices (BI), with BI=1.0 representing background noise. Thresholds for positive aAb signals were determined by applying the mathematical outlier criterion of P75+1.5-times IQR as indicated by a dotted line (**Figure 3**). According to these criteria, several samples with positive aAb were identified in all three tests in both control and patient group. Prevalence of natural S1PR1–aAb was 17.1% in SSc versus 2.7% in controls (**Figure 3A**). Similarly, prevalence of S1PR2–aAb was elevated with 19.0% in SSc patients versus 3.6% in controls (**Figure 3B**). In comparison of all three assays, S1PR3-aAb displayed the highest prevalence, with 8.3% in controls and 21.5% in patients, with one sample showing exceptionally high signal intensity with a BI of 13 (**Figure 3C**). A direct comparison of the prevalence for each of the three S1PR-aAb is provided in (**Figure 3D**) and highlights the statistically significant differences in S1PR-aAb prevalence in SSc versus controls.

### Prevalence of S1PR–aAb in Patients as a Function of Pulmonary Arterial Hypertension

**Figure 4:**
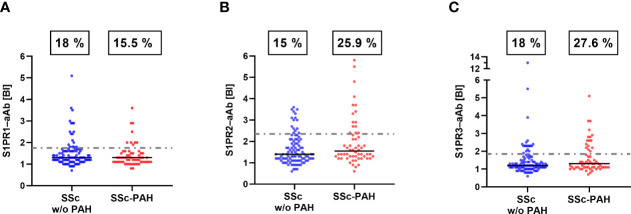
Prevalence of S1PR–aAb in SSc patients as a function of a diagnosis of PAH. SSc patients were subdivided into those with or without a diagnosis of PAH. **(A)** Prevalence of S1PR1-aAb was similar in both groups of patients, whereas **(B)** the prevalence of S1PR2-aAb was higher in SSc patients with PAH than in patients without PAH (P<0.05). **(C)** The difference in prevalence of S1PR3-aAb between both groups did not reach statistical significance (P>0.05). The binding indices (BI) along with the percentages of positive samples are provided. Prevalence was compared by one-sided Chi square test.

Pursuing the hypothesis that S1PR-aAb may be involved in the pathogenesis of PAH and therefore more prevalent in PAH, we subsequently divided the group of SSc patients into patients without PAH (SSc w/o PAH, n=100) and a smaller group of PAH-positive SSc patients (SSc-PAH, n=58). S1PR1-aAb appeared unrelated to PAH, and prevalence in the SSc w/o PAH group (18/100, 18.0%) was similar to the prevalence in the SSc-PAH group (9/58, 15.5%) (**Figure 4A**). In contrast, a quarter of samples in the SSc-PAH group displayed positivity for S1PR2-aAb (15/58, 25.9%), whereas a smaller percentage of SSc patients without PAH was positive for S1PR2-aAb (15/100, 15.0%), with the difference reaching statistical significance (P=0.0467) (**Figure 4B**). With regards to the prevalence of S1PR3-aAb, a higher fraction of samples from the SSc-PAH was identified as positive (16/58, 27.6%) in comparison to the SSc w/o PAH group (18/100, 18.0%), albeit without reaching statistical significance (P=0.1133) (**Figure 4C**). An overview of these three sets of measurements highlights the differential picture, with a particularly elevated prevalence of S1PR2-aAb in patients with SSc-PAH.

### Serum Samples With Positive Autoantibodies to More Than One S1PR

The samples showing positive autoimmunity were further analyzed with respect to recognizing more than one receptor, i.e., being positive in two or even in all three S1PR-aAb assays. In total, six positive samples were identified with aAb exceeding the threshold in all three S1PR-aAb assays, suggesting either a cross-reactivity of a single aAb to the different receptors or a polyclonal nature of the aAb within a given serum, with aAb co-existing and recognizing either S1PR1, or S1PR2 or S1PR3. All of the respective patients were female, five derived from the SSc cohort whereas only one healthy control was triple positive. Three of the triple positive subjects were suffering from SSc-PAH. Four samples were tested positive for both S1PR1-aAb and S1PR3-aAb. In addition, two SSc patient samples were simultaneously positive in the tests for S1PR1-aAb and S1PR2-aAb. A total number of 19 other samples were positive for S1PR2-aAb and S1PR3-aAb. The commercial antibodies yielded high signals only when tested for one receptor subtype, and not in the related assays.

### Comparison of Clinical Characteristics With Respect to S1PR–aAb

The full cohort of SSc patients was analyzed for clinical characteristics in relation to the results from the S1PR-aAb tests. In general, patients with S1PR3-aAb were younger than average (median age; 51 vs. 63). With respect to cutaneous manifestation of SSc, 85.2% of patients positive for S1PR1-aAb had a limited cutaneous form, whereas the diffuse manifestations were dominating in patients that were positive for S1PR2-aAb or S1PR3-aAb. Besides the elevated prevalence of S1PR2-aAb and S1PR3-aAb in the group of SSc patients with PAH, there was also a higher share of patients suffering from lung fibrosis in these groups (S1PR2-aAb; 60.7% vs. 45.3%, S1PR3–aAb; 71.9% vs. 41.9%). None of the groups showed remarkable differences in the prevalence of specific anti-nuclear Ab (**Table 2**).

**Table 2:** Characterization of the cohort of patients with systemic sclerosis regarding S1PR–aAb.

		S1PR1–aAb		S1PR2–aAb		S1PR3–aAb	
	SSc n=158	positive* n=27 (17.1%)	negative n=131 (82.9%)	positive* n=30 (19%)	negative n=128 (81%)	positive* n=34 (21.5%)	negative n=124 (78.5%)
**Cutanous manifestation**							
Limited, n (%)	91 (58%)	23 (85.2%)	68 (52.3%)	14 (48.3%)	77 (60.2%)	13 (39.4%)	78 (62.9%)
Diffuse, n (%)	59 (38%)	4 (14.8%)	55 (42.3%)	14 (48.3%)	45 (35.2%)	19 (57.6%)	40 (32.3%)
Sine scleroderma, n (%)	7 (4%)	0 (0%)	7 (5.4%)	1 (3.4%)	6 (4.7%)	1 (3.0%)	6 (4.8%)
mRSS, median (range)	6 (0–39)	4 (0–24)	6 (0–39)	5 (0–27)	6 (0–39)	5 (0–27)	6 (0–39)
**Pulmonary & cardiac involvement**							
NTproBNP [ng/L], median (range)	206 (5–19066)	296 (46–4884)	196 (5–19066)	135 (5–19066)	235 (29–14414)	135 (5–4884)	242 (29–19066)
PAH, n (%)	58 (37%)	9 (33.3%)	49 (37.4%)	15 (50%)	43 (33.6%)	16 (47.1%)	42 (33.9%)
ILD, n (%)	75 (48%)	11 (40.7%)	64 (49.6%)	17 (60.7%)	58 (45.3%)	23 (71.9%)	52 (41.9%)
Autoantibodies**							
anti-topoisomerase-1 (Scl70) Ab, n (%)	50 (33%)	4 (15.4%)	46 (36.2%)	11 (39.3%)	39 (31.2%)	16 (48.5%)	34 (28.3%)
anti-RNA-Pol-III Ab (ARA), n (%)	13 (8%)	2 (7.4%)	11 (8.7%)	2 (7.4%)	11 (8.7%)	4 (12.5%)	9 (7.4%)
anti-centromere-CENP-B Ab, n (%)	63 (41.4%)	21 (75%)	42 (33.6%)	11 (40.7%)	52 (41.6%)	9 (29.0%)	54 (44.6%)
anti-citrullinated-peptide Ab, n (%)***	30 (54.5%)	6 (66.7%)	24 (52.2%)	10 (71.4%)	20 (48.8%)	8 (53.3%)	22 (55%)

Missing values were excluded from the calculation; mRSS, modified Rodnan-Skin-Score; ILD, interstitial lung disease; *P75+1.5xIQR, **all were antinuclear Ab (ANA) positive, ***positive: ≥7 U/ml.

## Discussion

In this manuscript, we describe the establishment of novel analytical *in vitro* assays for assessing the prevalence of natural autoantibodies recognizing human S1PR1, S1PR2 or S1PR3. A first analysis on the specificity of the assays was positively passed by using commercial receptor-specific antibodies, recognizing only the respective receptor type and not cross-reacting to a related S1PR. Dependence of signals to antibody levels was observed in dilution experiments with commercial antibodies. As another important prerequisite for the present analysis, signal stability was shown by repeated freezing and thawing of aAb-positive human samples. In agreement with our hypothesis, prevalence of natural S1PR-aAb was significantly elevated in SSc patients as compared to healthy controls, with some further increase in the subgroup of patients who developed PAH. S1PR2-aAb and S1PR3-aAb showed a particular association with both PAH and lung fibrosis, in line with their established local biochemical function (39).

The balance between vasoconstriction and vasodilation in the (pulmonary) arterial system is fine-tuned by the expression of S1PR1, S1PR2 and S1PR3 on vascular endothelial cells and smooth muscle cells. While S1PR1 agonism in endothelial cells stimulates NO formation and causes vasodilation, S1PR2 and S1PR3 signaling in smooth muscle cells mediate vasoconstriction. Analyses in primary pulmonary artery smooth muscle cells, isolated perfused lungs and *in vivo* models of PH specifically identified signaling *via* S1PR2 to trigger pulmonary vasoconstriction and vascular remodeling (21, 22), while pharmacological inhibition of S1PR2 effectively prevented the development of chronic hypoxic PH in mice (9). Agonism of S1PR3, on the other hand, has been implicated in the development of radiation- and bleomycin-induced pulmonary fibrosis in mice (40, 41). The increased prevalence of S1PR2- and S1PR3- but not S1PR1-specific aAb in SSc-PAH patients may thus point towards an autoimmune-supported pathomechanism that could contribute to elevated pulmonary vascular resistance and the development of lung fibrosis in SSc-associated PAH. In line with this notion, PAH patients show elevated expression of S1PR2 on PASMC (9). Given that pharmacologic S1PR2 inhibition could prevent the development of hypoxic PH in rodents, an agonistic aAb against S1PR2/3 may conceivably promote PASMC proliferation and medial thickening (9). Yet, the biochemical nature of the S1PR-aAb and their functional effects on receptor signaling are presently unclear, as our newly developed assays only detect binding characteristics, while data probing whether the identified aAb act as antagonists to inhibit the receptors, as agonists to stimulate receptor signaling, or rather elicit allosteric effects are lacking and remain to be elucidated in future studies (40).

Our findings support earlier reports describing a set of GPCR-specific aAb in patients with autoimmune disease such as systemic sclerosis (28, 41). While aAb against a variety of GPCR including the endothelin type A receptor and angiotensin II receptor type 2 have been identified in a wide range of cardiovascular diseases where they have been proposed to contribute critically to disease initiation and/or progression, aAb against S1PR have so far only been reported in a single case report. In this lymphopenic patient with recurrent infections, aAb to human S1PR1 were identified (42). Adoptive transfer of the purified human aAb into mice caused immunosuppression, decreased T-cell chemotaxis and reduced lymph node egress *via* direct interaction with a subset of T-cells (42), thus highlighting the functional relevance of these aAb. The epitope recognized by the S1PR1-aAb was within the amino-terminal domain of S1PR1. Yet as therapeutic antibodies against certain S1PR demonstrated, binding is often not restricted to just one receptor (43). As such, it will be important to characterize the major antigenic epitopes for the specific receptor subtypes identified in the present study. Here, we describe for the first time the prevalence of S1PR-aAb including the first identification of S1PR2- and S1PR3-aAb in a large cohort of patients and healthy individuals, and report their potential use as both biomarkers and potential therapeutic targets in SSc, PAH, and/or lung fibrosis.

Among the particular strengths of our study are the rigorous assay designs, using full-length human receptor molecules as bait and human cells as system for the recombinant expression, thereby enabling regular posttranslational modifications, full coverage of potential epitopes and correct trafficking (44, 45). Moreover, the identification of commercial receptor-specific antibodies to each of the three receptor subtypes verified specificity of the assays, and enables other research groups to conduct similar analyses using the same type of positive controls, thereby increasing transparency and comparability between analytical techniques. The cohort size of the controls and of the patients provided considerable numbers of samples, allowing for a solid case-control comparison and a focused analysis of patients with versus without PAH. Finally, assay sensitivity was high and only small amounts of serum were required to obtain the aAb result, enabling fast, cost-effective and high throughput analyses in future studies on larger cohorts of samples with related cardiovascular phenotypes.

No study is without limitation, and the lack of biological characterization of the identified aAb as potential agonists, antagonists or modulators of receptor function constitutes an important knowledge gap. Unfortunately, sample volumes were limited and not sufficient to isolate immunoglobulins for further biochemical analyses. The nature of the study design as case-control study precludes any causal interpretations, and detailed structural and functional analyses of the identified aAb along with analyses with longitudinal patient samples and clinical data are required to elucidate a potential etiologic role of the aAb in SSc, and in particular in PAH. In addition, information on anthropometric and clinical characteristics of the participants contributing to the cohort of healthy subjects is sparse, as the samples were obtained from a commercial supplier, and subject-specific information was not provided for reasons of data safety. Finally, genetic background and place of residency of the patients was relatively uniform, precluding extrapolations to patients from other populations or different genetic background. That notwithstanding, this is the first systematic analysis of aAb to human S1PR in healthy and diseased subjects, providing some positive results in relation to PAH which warrant further analysis in larger clinical studies.

## Data Availability Statement

The original contributions presented in the study are included in the article/supplementary material. Further inquiries can be directed to the corresponding authors.

## Ethics Statement

The studies involving human participants were reviewed and approved by Charité Medical School Berlin (10/30/2017, EA1/178/17) and the Board of Ethics of the University of Cologne (#04-037). The patients/participants provided their written informed consent to participate in this study.

## Author Contributions

Conceptualization, ES, SS, and LS; Methodology: HG, WM, and JH; Software: HG and JH; Validation, SS, GR, and WK; Formal Analysis: HG, JH, SS, and LS; Investigation: HG, ES, WM, SS, and LS; Resources: ES, WM, GR, and LS; Data Curation: HG, JH, SS, and LS; Writing—Original Draft Preparation, HG, ES, SS, and LS; Writing, Review and Editing; WM, JH, GR, and WK; Supervision, SS and LS; Project Administration, LS; Funding Acquisition, LS. All authors have read and agreed to the published version of the manuscript.

## Funding

The research was supported by the Deutsche Forschungsgemeinschaft (DFG), research unit FOR-2558 “TraceAge” (Scho 849/6-2), and CRC/TR 296 “Local control of TH action” (LocoTact), to LS, and SFB-TR84 and KU1218/12-1 to WK. Additional support was received from the German Centre for Cardiovascular Research (DZHK) and German Foundation for Heart Research (F-09-19) to SS. ES was supported by the Berlin Institute of Health Biomedical Innovation Academy with a Fellowship of the BIH Charité Clinician Scientist Program.

## Conflict of Interest

The authors declare that the research was conducted in the absence of any commercial or financial relationships that could be construed as a potential conflict of interest.

## Publisher’s Note

All claims expressed in this article are solely those of the authors and do not necessarily represent those of their affiliated organizations, or those of the publisher, the editors and the reviewers. Any product that may be evaluated in this article, or claim that may be made by its manufacturer, is not guaranteed or endorsed by the publisher.
